# The clinical value of color ultrasound screening for fetal cardiovascular abnormalities during the second trimester: A systematic review and meta-analysis

**DOI:** 10.1097/MD.0000000000034211

**Published:** 2023-07-14

**Authors:** Bingye Shi, Zhe Han, Wei Zhang, Wenxiu Li

**Affiliations:** a Color Ultrasound Room, Affiliated Hospital of Hebei University, Baoding, China; b Department of Cardiac Surgery, Affiliated Hospital of Hebei University, Baoding, China; c Medical Engineering Center, Affiliated Hospital of Hebei University, Baoding, China; d Beijing Anzhen Hospital, Capital Medical University, Beijing, China.

**Keywords:** cardiac abnormality, color doppler, meta-analysis, second trimester

## Abstract

This meta-analysis aimed to investigate the diagnostic value of color ultrasound screening for fetal cardiovascular abnormalities during the second trimester in China.

**Methods:**

A literature search was conducted to evaluate the clinical value of color ultrasound screening for fetal cardiovascular abnormalities during the second trimester using English and Chinese databases. Numerical values for sensitivity and specificity were obtained from false-negative, false-positive, true-negative, and true-positive rates, presented alongside graphical representations with boxes marking the values and horizontal lines showing the confidence intervals. Summary receiver operating characteristic (SROC) curves were applied to assess the performance of the diagnostic tests. Data were processed using Review Manager 5.3.

**Results:**

Four studies (151 patients with fetal cardiovascular abnormalities and 3397 undiagnosed controls) met our evaluation criteria. The sensitivity was 0.91 and 0.96, respectively, and the specificity was 1.00. The Area Under the Curve (AUC) from the SROC curves was >90%; therefore, it was classified as excellent. Furthermore, there were 6 types of fetal cardiovascular abnormalities, and the pooled screening rate of atrioventricular septal defects was the highest.

**Conclusion:**

Our meta-analysis showed that the use of color ultrasound during the second trimester can be an excellent diagnostic tool for fetal cardiovascular abnormalities.

## 1. Introduction

Congenital heart abnormalities are the most common structural abnormalities associated with fetal cardiovascular development.^[1]^ An abnormal fetal cardiovascular function affects fetal health. The rate of congenital heart abnormalities is approximately 0.8% in fetuses, which reduces the population birth quality.^[2]^ The embryo begins to form a cardiovascular system at approximately 3 weeks of pregnancy but is not able to circulate blood independently.^[3]^ The internal structure of the fetal heart does not completely develop until 7 to 8 weeks of pregnancy.^[4]^ The exposure of pregnant women to external adverse stimulation during fetal cardiovascular system development at 7 to 8 weeks of pregnancy may hinder its proper development. In severe cases, the fetus is delivered with congenital heart disease, which severely affects the fetal quality of life in the later years.^[5,6]^

The causes of fetal cardiovascular abnormalities include heredity factors, fetal factors, and the maternal environment.^[7]^ Older women infected with viruses, or those who have congenital diseases, are more likely to give birth to newborns with fetal cardiovascular abnormalities.^[7]^ Furthermore, abnormalities in fetal chromosomes and amniotic fluid, and developmental delay in utero can also lead to cardiovascular abnormalities.^[8]^ After the birth of a newborn, its symptoms are not obvious and will delay detection time, resulting in increased difficulty in treatment.^[8]^ Due to insufficient development of the fetal cardiovascular system in early pregnancy, there may be some deviation in the detection results of fetal heart disease by ultrasound examination. In the second trimester of pregnancy, fetal orientation and fetal heart development are relatively stable, so it may be the best time to explore fetal heart development.^[9]^

Color Doppler is the use of autocorrelation technology for Doppler signal processing, and the blood flow signal obtained by *autocorrelation* technology is color-coded and then superimposed on a 2-dimensional image in real time, leading to the formation of a color Doppler blood flow image.^[10]^ It offers several advantages, including minimal trauma, no radiation exposure, quick and convenient operation, and widespread utilization in clinical practice.^[11]^ It can be used for examination of the brain,^[12]^ liver,^[13]^ spleen,^[14]^ stomach,^[15]^ kidney,^[16]^ breast^[17]^ and uterus,^[18]^ along with screening for vascular diseases, especially cardiovascular diseases.^[19]^

Color Doppler can be conveniently and accurately applied in the screening of fetal heart malformations during pregnancy, which can clearly diagnose and reduce the birth rate of newborns with congenital heart disease.^[20]^ Color Doppler can be used to assess the intrauterine placenta, umbilical cord, and amniotic fluid status of pregnant women. Furthermore, it can clearly show the general structure of the fetus in utero and the 4 chambers of the heart. It aids in clear visualization of the cardiac vein, artery abnormality, ventricular outflow tract, blood vessels, and other conditions, leading to an improved detection rate.^[20]^ This is of great significance for high-quality fertility.

Color Doppler has been widely used in prenatal fetal screening for cardiovascular abnormalities in China because of its convenience, low trauma, and lack of radiation. Several studies have evaluated the clinical compliance rate of color Doppler in screening for fetal cardiovascular abnormalities during pregnancy.^[21–24]^ However, these studies were conducted with small sample sizes and were not representative of the general Chinese population. In addition, there have been few meta-analyses on the diagnostic accuracy of color Doppler in the diagnosis of fetal cardiovascular abnormalities during the second trimester in the Chinese population. Therefore, the purpose of this study was to use a meta-analysis to comprehensively evaluate these studies and the types of cardiovascular abnormalities in the Chinese population.

## 2. Materials and Methods

### 2.1. Study search

A systematic review was conducted according to the methods and recommendations of the PRISMA extension statement for reporting systematic reviews. The Cochrane Collaboration Systematic Reviews of Diagnostic Test Accuracy handbook was also consulted. We identified studies published in English between the years 2015 and 2022. English language (PubMed, Google Scholar, Cochrane Library, and Clinical Trials) and Chinese language databases (CNKI, Cqvip, WANFANG data, and Baidu scholar) were searched applying controlled vocabulary (MeSH keywords), as well as using the terms “cardiac abnormality,” “heart anomaly,” “heart disease,” “congenital,” “Color Doppler,” “second trimester,” “China,” and “Chinese,” or variants and combinations of these keywords. The reference lists of the included studies were reviewed to identify other potentially eligible studies.

### 2.2. Exclusion criteria

The selected publications were independently evaluated by 2 reviewers based on the established inclusion criteria. These publications were selected based on the information provided in the title and/or abstract as well as their full text, if available in English. Furthermore, these publications were studies on Color Doppler in fetal cardiovascular abnormalities in China. The samples used in these studies were human specimens. Additionally, the samples in these publications were diagnosed using 2-stage screening (a questionnaire for primary screening and formal diagnosis by physicians according to the conventional diagnostic criteria in China). The following studies were excluded from this systematic review and meta-analysis: non-research-based publications, such as reviews, press releases, newsletters, and forums and unclear diagnostic criteria or unconventional diagnostic tools.

### 2.3. Data extraction and quality assessment

The corresponding data were extracted from studies that met the inclusion criteria and contained the following information: first author, year of publication, country, and sample size (including the number of positive and negative groups). Indicators such as sensitivity and specificity and number of true positives, false positives, false negatives, and true negatives were recorded directly or calculated indirectly according to the original data of the enrolled studies. In addition, data on the disease types of the positive patients were extracted and collected. The quality of the selected publications was estimated following the criteria of the Grading of Recommendations Assessment, Development, and Evaluation method. The quality of the enrolled studies was assessed using the Cochrane Center Quality Assessment of Diagnostic Accuracy Studies (QUADAS-2).

### 2.4. Statistical analysis

The meta-analysis was performed using Review Manager 5.3 (Copenhagen: The Nordic Cochrane Centre, The Cochrane Collaboration, 2014). Forest plots were used to summarize the estimates with 95% confidence intervals. The random-effects model was used to calculate the pooled sensitivity, specificity, positive predictive value, and negative predictive value. The summary receiver operating characteristic (SROC) curve was plotted and the area under the curve (AUC) of the SROC was then calculated. A rough guide for classifying the accuracy of a diagnostic test was based on the AUC. The criteria for AUC classification were 90% to 100% (excellent), 80% to 90% (good), 70% to 80% (fair), 60% to 70% (poor), and 50% to 60% (failure).^[25]^

For fetal cardiac anomaly studies, a random-effects model was used. The meta-analysis was performed using Review Manager 5.3. The screening rate was computed for each study to derive a pooled screening rate estimate using the inverse variance method, which involved calculating the weighted average using SEs. The variance of the screening rate of each estimate (known as effect size) was calculated as sqrt (pq/n), where p is the screening rate, q is 1-p, and n is the total sample size. Heterogeneity was anticipated in advance, and Cochrane Q test and Higgins I^2^ statistics were used. The I^2^ test was also used to assess the heterogeneity between studies (I^2^ = 75%–100%, *P* < .05). Forest plots were used to summarize the estimates with 95% confidence intervals.

## 3. Results

### 3.1. Description of studies

**Figure 1.**
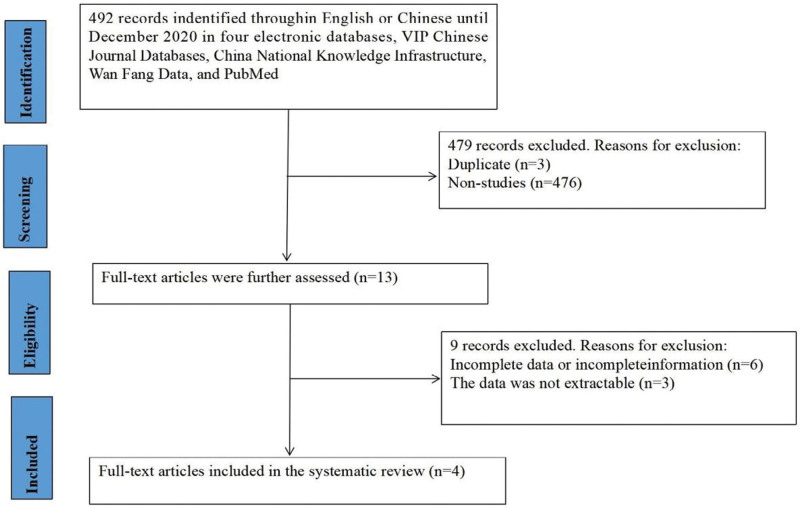
Flow chart of the study selection process.

Based on the database search strategies, 492 Chinese or English articles were identified. Thirteen full-text articles were selected, and duplicate citations and studies irrelevant to the current meta-analysis were excluded. After excluding 9 articles with incomplete data, 4 met the inclusion criteria and were included in the systematic review (Fig. 1). All 4 articles were Chinese (Table 1). A total of 151 patients diagnosed with fetal cardiovascular abnormalities after delivery, and 3397 undiagnosed controls were included in the 3 studies. The basic characteristics of the included studies are presented in Table 1.

**Table 1:** Characteristics of studies included in the present meta-analysis.

Reference	Yr	Country	Patients/controls	Method	TP	FP	FN	TN	Sensitivity (95% CI)	Specificity (95% CI)
Guohuai Zhang^[11]^	2019	China	47/1433	Color Doppler	47	1	2	1433	96 (86,100)	100 (100, 100)
Haijing Fan^[12]^	2020	China	20/234	Color Doppler	20	1	1	234	95 (76,100)	100 (98, 100)
Haohan Li^[13]^	2015	China	74/2926	Color Doppler	74	8	4	2926	95 (87, 99)	100 (99,100)
Jing Chen^[14]^	2020	China	10/190	Color Doppler	10	0	1	190	91 (59, 100)	100 (98, 100)

FN = false negative, FP = false positive, TN = true negative, TP = numbers of true positive.

### 3.2. Publication bias

**Figure 2.**
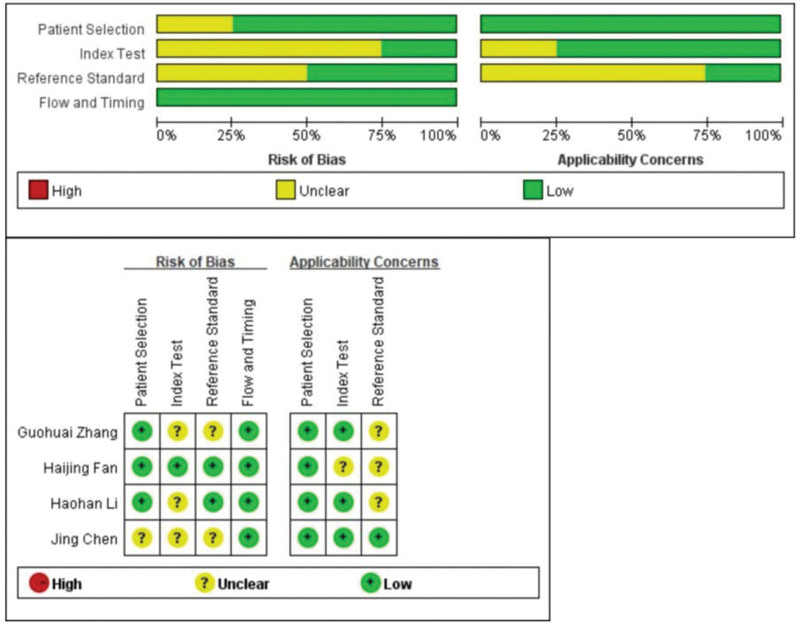
Quality of selected studies according to QUADAS-2 guidelines.

The QUADAS-2 tool was used to assess the quality of the included studies (Fig. 2). We evaluated the quality of the diagnostic studies based on the QUADAS-2 criteria in 6 key areas: patient selection, index testing, reference criteria, flow and timing, judgment bias, and applicability. Each area was assessed in terms of risk of bias, and the first 3 areas were assessed in terms of suitability. The outcome of the assessments could be “yes,” “no,” or “unclear” for each item. A “yes” implies a lower risk of bias, while a “no” or “unclear” outcome implies the opposite (Fig. 2).

### 3.3. Analysis of diagnostic accuracy

**Figure 3.**
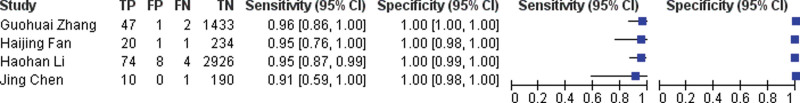
Forest Plot of Color Doppler for the diagnosis of fetal cardiovascular abnormalities.

**Figure 4.**
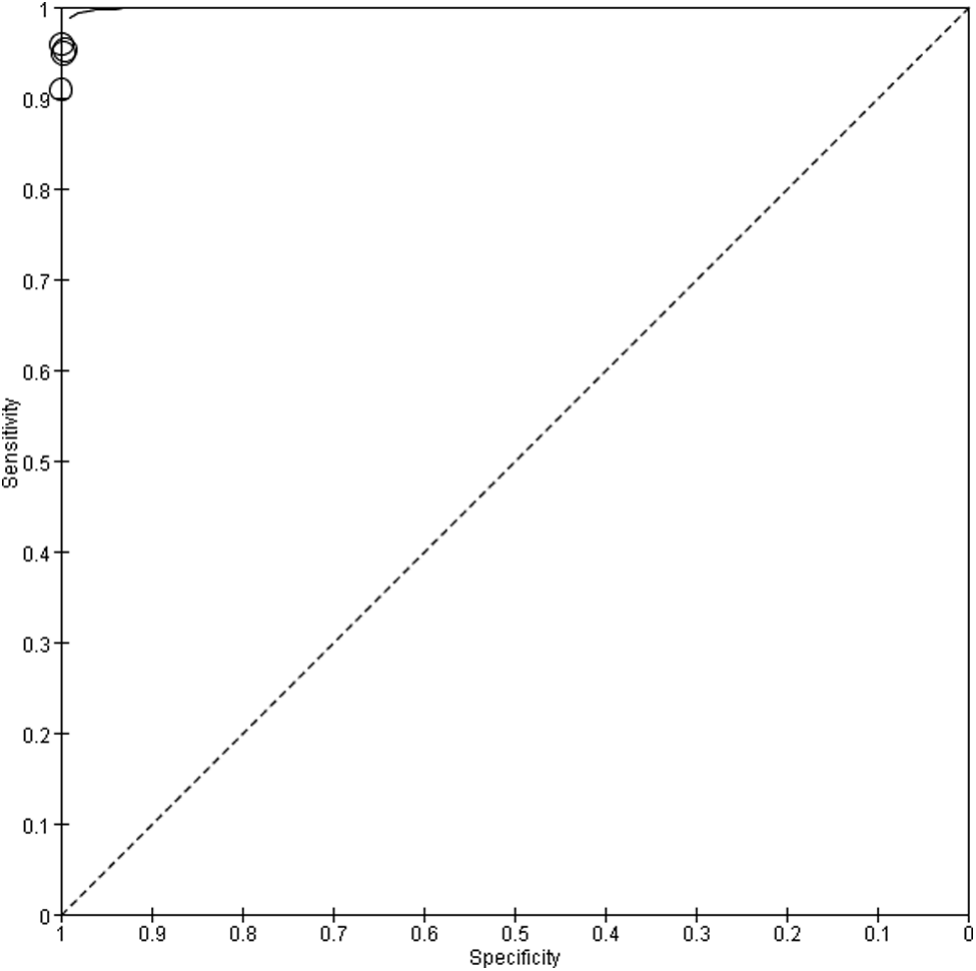
SROC Plot for Color Doppler in the diagnosis of fetal cardiovascular abnormalities. SROC = summary receiver operating characteristic.

The results of the diagnostic accuracy analysis showed that the sensitivity value was between 0.91 and 0.96, and the specificity value was 1.00 (Table 1 and Fig. 3). An SROC plot based on the results of this meta-analysis was constructed, and the pooled AUC of the SROC was found to be >90% (Fig. 4).

### 3.4. Analysis of types of fetal cardiac anomaly

**Figure 5.**
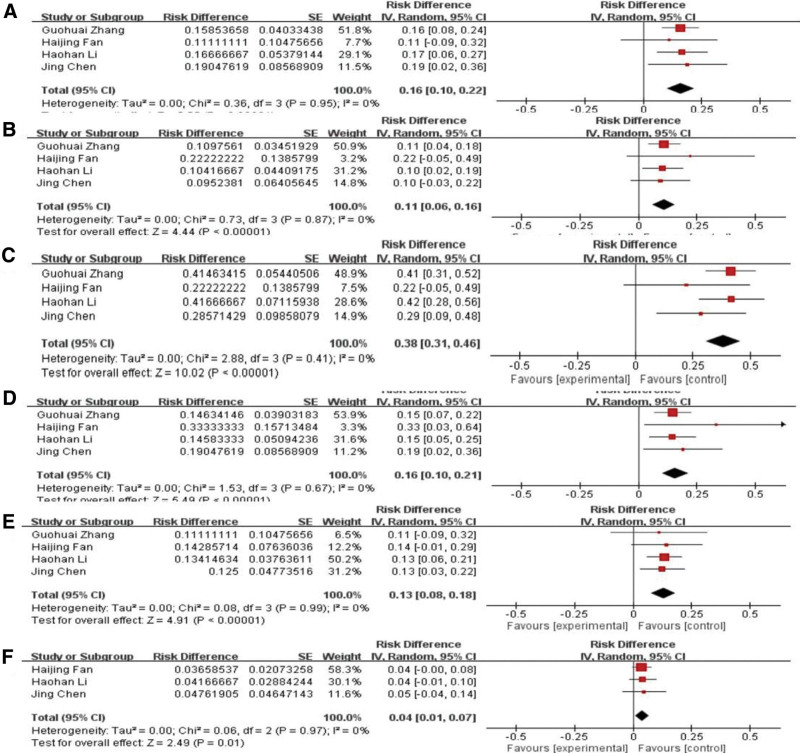
Analysis of types of fetal cardiac anomaly (A–F): tricuspid atresia, tetralogy of Fallot, atrioventricular septal defect, single ventricle, ventricular septal defect, and single atrium.

As shown in Figure 5, there were 7 types of fetal cardiovascular abnormalities according to the type distribution: tricuspid atresia, tetralogy of Fallot, atrioventricular septal defect, single ventricle, ventricular septal defect, and single atrium. Screening rates were 16%, 11%, 36%, 16%, 13%, and 4%, respectively. The pooled screening rate of endocardial pad defects was the highest and was significantly different from that of the other types (*P* < .05).

## 4. Discussion

Color ultrasound is suitable for clinical applications because it poses little trauma, is convenient, and lacks radiation.^[26]^ Color Doppler examination for pregnant women between 20 and 28 weeks of pregnancy is performed mainly to detect any cardiovascular abnormalities in the fetus. This information is then used to determine whether to induce labor surgically or to continue the pregnancy, which can help avoid fetal malformation to an extent and ensure the quality of the newborn.^[27]^ It is important to screen for fetal heart malformation during pregnancy. If cardiovascular abnormalities are detected on time during the fetal period, the birth of such fetuses can be avoided.^[28,29]^ Screening for fetal cardiovascular abnormalities using prenatal color ultrasound can provide a basis for pregnant women to continue or terminate pregnancy, thus helping to reduce the number of newborns with congenital heart diseases and improve the eugenic birth rate of the population.

Color ultrasound was developed based on B-ultrasound. It has multiple functions, multiple diagnostic methods, high accuracy, a wide examination range, and specific content. Studies have shown that the detection rate of congenital cardiovascular abnormalities using color ultrasound is higher than that of B-ultrasound.^[20]^ Color ultrasound not only provides clear images intuitively but also monitors the growth process of the fetus and checks the amniotic fluid condition in the womb and placental umbilical cord.^[30]^ In addition, color ultrasound can clearly show the structure of the fetal body, 4-chamber surface of the heart, vascular conditions, ventricular outflow tract, and arteriovenous abnormalities. This is beneficial for improving the diagnostic rate.^[31]^ The results of this study showed that the pooled AUC of the SROC was >90%, indicating excellent diagnostic value. This result also supports the clinical application of color ultrasound.

The fetal cardiovascular system takes shape in the early stage; however, it cannot circulate blood effectively at this stage and is easily affected by fetal orientation.^[32]^ After the second trimester, the heart structure is formed and is not affected by fetal orientation. Therefore, the second trimester is the best time to screen for fetal cardiovascular abnormalities. The second trimester was selected as the screening time for the 11 included studies. A systematic review showed that the sensitivity and specificity of color ultrasound in the diagnosis of fetal cardiovascular disorders in the first trimester were 55.80% and 99.98%, respectively, and suggested that the introduction of color Doppler in early ultrasound examination of fetal heart can improve the detection rate of f fetal cardiac pathology.^[2]^ By meta-analysis of the present study, we found that the pooled AUC of SROC was >90% in the diagnosis, the sensitivity value was between 0.91 and 0.96, and the specificity value was 1.00. These results suggest that the diagnostic effect of color ultrasound in the second trimester may be more accurate.

Fetal cardiovascular abnormalities are mainly divided into 2 categories: nonstructural cardiovascular abnormalities, mainly caused by the complete development of the embryo, and structural cardiovascular abnormalities, mainly caused by embryonic development.^[33]^ The results of this study showed that in the 4 included studies, the fetuses were all characterized by structural cardiovascular abnormalities, and the screening rate of atrioventricular septal defects was the highest among all types. The reason why this type of exposure has the highest rate in patients with fetal cardiovascular abnormalities requires further analysis.

The accurate evaluation of cardiac function is crucial for the early diagnosis of cardiovascular disease. Although this study found that color Doppler in the second trimester had an acceptably high accuracy in diagnosing fetal cardiovascular abnormalities. However, diagnostic techniques must be improved to improve diagnostic efficiency and accuracy. A video-based deep-learning algorithm, EchoNet-Dynamic, has outperformed human experts in segmenting the left ventricle, estimating ejection fraction, and evaluating diagnostic manifestations of cardiomyopathy.^[34]^ This study reveals the importance of deep learning in automatically assessing cardiac function from echocardiographic videos. EchoNet-Dynamic can improve the precision and accuracy of existing methods, allowing earlier detection of subclinical cardiac dysfunction. Additionally, DPS-Net, a 2-dimensional echocardiogram based on deep learning, reportedly automatically measures the left ventricular ejection volume.^[35]^ The combined evaluation of large-scale data sets shows that DPS-Net is highly adaptable in different echocardiographic systems. All of these have laid the foundation for the future development of imaging diagnosis of fetal cardiovascular abnormalities.

A limitation of this study is that in terms of diagnostic accuracy, only 4 studies met the inclusion criteria, and the diagnosis process was not described in detail after fetal induction or delivery, which may have affected the overall estimation. In addition, this meta-analysis included studies involving only the Chinese population; therefore, there may be some limitations to the generalization of the conclusions. Moreover, only papers published in Chinese were included, and papers in other languages were excluded, leading to inevitable errors. Nonetheless, our analysis shows that color ultrasound examination in the second trimester of pregnancy can be used as a clinical diagnostic tool for the diagnosis of fetal cardiovascular abnormalities. However, owing to the limitations of this study, high-quality, large sample sizes from multiple centers are still needed for further validation.

## 5. Conclusions

The use of color ultrasound by pregnant women during the second trimester is a good diagnostic tool for fetal cardiovascular abnormalities. In addition, this diagnostic method can be used for early diagnosis of pregnant women in advance, which contributes to high quality fertility.

## Author contributions

**Conceptualization:** Wenxiu Li.

**Data curation:** Bingye Shi, Zhe Han.

**Methodology:** Bingye Shi, Zhe Han.

**Software:** Bingye Shi, Zhe Han.

**Validation:** Wei Zhang, Wenxiu Li.

**Writing – original draft:** Wenxiu Li.

**Writing – review & editing:** Wei Zhang, Wenxiu Li.
